# Supplementary material for: Analysis of PPARγ Signaling Activity in Psoriasis
Source: Int J Mol Sci. 2021 Aug 10;22(16):8603. doi: 10.3390/ijms22168603 (PMC8395241; doi:10.3390/ijms22168603)
Supplement: Supplementary file 1 [file ijms-22-08603-s001.zip › Supplemental materials_Analysis of PPARg signaling activity in psoriasis/Pathway models/Models images and html files/Model of PPARG related pathways in psoriasis (short version)/50532.html]

cAMP


# Small Molecule cAMP

|  |  |
| --- | --- |
| URN | urn:agi-cas:60-92-4 |
| Total Entities | 13 |
| Connectivity | 9838 |
| Name | cAMP |
| Class | Endogenous compound |
| Molecular Weight | 329.205941 |
| XLogP | -2.600000 |

---

|  |  |
| --- | --- |
| ChildConcepts | 8-Br-cAMP |
|  | 8-CPT-cAMP |
|  | 8-thiomethyl-cAMP |
|  | 8-benzylthio-cAMP |
|  | 8-Cl-cAMP |
|  | 8-Br-cGMP |
|  | 3'NH-cAMP |
|  | 8-N3-cAMP |
|  | 8-pCPT-cGMP |
|  | N6-benzyl-cAMP |
|  | 8-AHA-cAMP |
|  | 2'-deoxy-cAMP |
|  | cGMP |

---

|  |  |
| --- | --- |
| Pathway | Circadian Clock Genes Mutations Cause Insomnia |
|  | Melatonin Effects on Circadian Cycle |
|  | mTOR Signaling |
|  | Hypoxia Inhibits mTOR Signaling |
|  | High Fat Diet Activates cAMP Related Exocytosis in Synapse and Endocrine Cell |
|  | GCG and PPY Regulate Metabolism and Satiety |
|  | GUCY2C in Intestinal Ion and Fluid Homeostasis |
|  | Ghrelin and Nesfatin-1 Regulate Stomach Functional State and Satiety |
|  | mTOR Signaling Activation by Taste Sensor Receptors (Rodent Model) |
|  | Ghrelin and SST Regulate GH1 Release in Hypothalamus |
|  | L-cell: GCG, PYY and 5-HT Release |
|  | P-cell Activation |
|  | K-cell: GIP Production |
|  | I-cell: CCK-8 Secretion and Regulation of Eating Behavior |
|  | D-cell: Somatostatin Release |
|  | M-cell: Motilin Release |
|  | A-cell (X-like): Ghrelin and Nesfatin-1 Synthesis |
|  | G-cell: Gastrin Secretion |
|  | ECL-cell: CHGA and Histamine Synthesis |
|  | N-cell: Neurotensin Production |
|  | Insulin Synthesis in beta-Cell |
|  | Insulin Release Inhibition by Somatostatins |
|  | Dopamine Receptors Signaling |
|  | Serotonin Receptors Signaling |
|  | Catecholamines Secretion from Adrenal Gland |
|  | Circadian Clock and Sleep Regulation by Melatonin |
|  | Norepinephrine Release Regulation |
|  | Dopamine Mediated Glutamate Release/Uptake Circle in Neuron in Migraine |
|  | Dopamine Synthesis and Release |
|  | CRH Secretion Regulation |
|  | Insulin Signaling |
|  | Prolactin Production |
|  | Somatostatin Receptors Signaling |
|  | POMC Secretion in adenohypophysis |
|  | Testicular AMH Production |
|  | LH and FSH Signaling and Steroidogenesis |
|  | Estrogens in Prolactin Production |
|  | Mineralcorticoids Secretion Regulation |
|  | Steroid Hormones Steroidogenesis |
|  | CXC Chemokine Receptor Signaling |
|  | CC Chemokine Receptor Signaling |
|  | Proplatelet Maturation |
|  | Platelet Activation via GPCR Signaling |
|  | Hypothalamic-Pituitary-Adrenal Axis in Acute Phase Response |
|  | MC1R in anti-Inflammatory Signaling |
|  | Prostaglandin E2 Receptor Signaling in Neurons |
|  | Leukotriene Effect on Vascular Endothelial Cell Response |
|  | Eicosanoids in Myeloid Cell Activation |
|  | Histamine Receptor H3 Signaling in Neurons |
|  | Prostaglandin E2 and Histamine in Gastric Acid Secretion |
|  | Neutrophil Activation via LTB4/Polyisoprenyl Phosphate Signaling |
|  | Basophil Activation |
|  | Mast-Cell Activation without Degranulation through CRHR1 Signaling |
|  | Mast-Cell Activation without Degranulation |
|  | Mast-Cells De Novo Synthesized Mediators via IgE Independent Signaling |
|  | Neutrophil Chemotaxis |
|  | SNS Related Adrenal Cortex Response |
|  | Vascular Endothelial Cell Activation by NO |
|  | Vasodilation Activation |
|  | Vascular Endothelial Cell Activation by Cytokines |
|  | Lipolysis Regulation in Adipocyte |
|  | Synaptic Potentiation by PKMZ in LTP Maintenance - Active State |
|  | Potassium Flux Regulation |
|  | Nociception Expression Targets Signaling |
|  | Sodium Flux Regulation |
|  | Vascular Motility |
|  | Ca2+ Flux Regulation |
|  | TAAR1 -> Neurotransmitter Uptake |
|  | NPY1R -> CRH/POMC Production |
|  | CGRP -> Calcium Influx |
|  | CRH -> Synthesis of Corticosteroids |
|  | EDNRA/B -> Vascular Motility |
|  | ADORA2A/B -> Vasodilation |
|  | ADORA1/2A -> Exocytosis |
|  | ADRB1/3 -> Vasodilation |
|  | ADRA1 -> Ion Channels |
|  | ADRA2C/ADRB2 -> Vasoconstriction |
|  | ADRB1 -> Ion Channels |
|  | ADRA2C/ADRB2 -> Synaptic Endocytosis |
|  | ADRA2A -> Hyperpolarization |
|  | ADRB1 -> Prostaglandin Generation |
|  | BDKRB1/2 -> Vasodilation |
|  | BDKRB1/2 -> Ion Channels |
|  | CNR1/2 -> Membrane Transport |
|  | CNR1/2 -> Vascular Motility |
|  | CNR1/2 -> IL1B/2/4/6/10 Production |
|  | DRD1/5 Expression Targets |
|  | DRD2/4 -> Membrane Transport |
|  | GALR1/2/3 -> POMC/NPY Production |
|  | GALR1/2/3 -> Neurotransmitter Metabolism |
|  | HRH1/2 -> Membrane Polarization |
|  | HRH1/3 -> Synaptic Transmission |
|  | HRH1/2 -> Vascular Motility |
|  | HRH2/4 -> IL6/10 Production |
|  | CHRM1/2/3/5 -> Ion Channels |
|  | CHRM1/2/3 -> Vascular Motility |
|  | OPRD/OPRM -> Ion Channels |
|  | OPRK -> Pain Perception |
|  | OPRL1 -> Ion Channels |
|  | PTGIR -> IL6 Production |
|  | PTGDR -> Vasodilation |
|  | PTGER1/4 -> Vascular Motility |
|  | PTGER2/3 -> Vascular Motility |
|  | PTGIR -> Vasodilation |
|  | HTR4/6/7 -> Vasodilation |
|  | HTR7 -> IL6 Production |
|  | HTR4/6/7 -> Cation Channels |
|  | HTR2 -> Membrane Transport |
|  | HTR1 -> Vascular Motility |
|  | TACR1 -> Membrane Transport |
|  | CHRNA7 -> NOS1 Production |
|  | GABA(B)R -> Postsynaptic Inhibition |
|  | GRM2-4/6-8 (Presynaptic) -> Glutamate Release Attenuation |
|  | GRM1/5 (Postsynaptic) -> Ion Channels |
|  | P2RY11/13/14 -> IL8/10 Production |
|  | Luteinizing Hormone in Oocyte Meiotic Resumption |
|  | GPR3 in Oocyte Meiotic Arrest |
|  | Agomelatine Antidepressant Action |
|  | Histamine in Arousal Regulation |
|  | Endocannabinoids Role in Sleep Regulation |
|  | Circadian Clock Genes in Suprachiasmatic Nuclei Neurons |
|  | Prostaglandins Role in Sleep Regulation |
|  | HRH3 in Negative Regulation of Histamine Production and Release in Sleep Regulation |
|  | Adenosine Role in Sleep Regulation |
|  | Retinal Ganglion and Suprachiasmatic Nucleus in Melatonin Synthesis Regulation |
|  | Melatonin Synthesis in Pinealocytes |
|  | Vitamine D Deficite and Dentin Formation |
|  | non-Genomic Rapid Actions of Vitamin D in Vitamin D Biology |
|  | Gap Junction Assembly |
|  | beta-Cell Granules Exocytois |
|  | Cardiomyocyte Dysfunction |
|  | Dilated Cardiomyopathy |
|  | Alcoholic Dilated Cardiomyopathy (Mouse Model) |
|  | Ca2+ Signaling in Dilated Cardiomyopathy |
|  | Angiotensin-Aldosterone System Activation Scheme |
|  | Arterial Hypertension |
|  | Aldosterone Synthesis Acute Regulation |
|  | Endothelial Cell Dysfunction in Pulmonary Hypertension |
|  | Vascular Reactivity in Raynaud Disease |
|  | NO Synthesis in Early Vasospasm |
|  | Contraction Due Vasospasm |
|  | Sebocyte Proliferation in Acne Vulgaris |
|  | Hyperseborrhea in Acne Vulgaris |
|  | Androgens Synthesis in Sebocytes |
|  | Melanoma |
|  | MITF as a Regulator of Melanoma Cell Development |
|  | Melanocyte Role in Skin Aging |
|  | Wolfram Syndrome Progression (Hypothesis) |
|  | Leptin in Insulin Synthesis and Secretion |
|  | beta-Cell Destruction through Cytokines in Diabetes Mellitus |
|  | beta-Cell Mass Regulation |
|  | beta-Cell Recovery in Diabetes Mellitus |
|  | G-Proteins Signaling in Insulin Secretion in beta-Cell |
|  | beta-Cell Function Inhibition by Ciclosporine and Tacrolimus (Rodent Model) |
|  | Ghrelin Influence on Insulin Secretion (Hypothesis) |
|  | Ca2+ Reabsorption Decline in Intestine |
|  | Bone Resorption in Hyperparathyroidism |
|  | Ca2+/Pi Reabsorption Decline in Kidney in Hyperparathyroidism |
|  | Hypothyrodism Associated Hyperprolactinemia |
|  | DRD2 Pharmacological Inhibition in Hyperprolactinemia |
|  | Ethanol Induced Efects in Hyperprolactinemia |
|  | Bone Remodeling in Hyperthyroidism |
|  | Cardiovascular Effects in Hyperthyroidism |
|  | Thyroid Dysfunction |
|  | Graves Ophthalmopathy |
|  | GNRH Signaling Impairment in Hypogonadotrophic Hypogonadism |
|  | Ca2+/Pi Reabsorption Decline in Kidney in Hypoparathyroidism |
|  | Osteoblast Dysfunction in Hypoparathyroidism |
|  | Hypothyroidism, Secondary (Central) |
|  | Thyroid Stimulating Hormone (TSH) Resistance in Congenital Hypothyroidism |
|  | Overt Hypothyroidism, Primary |
|  | Thyrotropin Releasing Hormone (TRH) Hypothalamic Release in non-Autoimmune Hypothyroidism |
|  | Thyroid Stimulating Hormone (TSH) Secretion in Overt Hypothyroidism |
|  | Central Hypothyroidism |
|  | Tertiary Hypothyroidism |
|  | SOCS3 and PTPN1 in Hypothalamic Neuron Insensitivity to Insulin and Leptin |
|  | Prolactinoma Overiew |
|  | Metastatic Colorectal Cancer |
|  | Basophil Activation in Asthma |
|  | CFTR Facilitates Oxidative Stress in Airway Epithelium in CF |
|  | Airway Epithelial Cell Dysfunction |
|  | CFTR in Sperm Capacitation and Acrosome Reaction |
|  | CFTR Expression in Epithelial Cells (Class I Mutations) |
|  | CFTR Related Ion-Channel Dysfunction in Cystic Fibrosis Airway Epithelium (Class III Mutations) |
|  | Osteoblast Function Decline in Gout |
|  | Osteoclast Activation in Gout |
|  | Steroidogenesis Impairement in Polycystic Ovary Syndrome |
|  | Ovulation Block |
|  | Polycystic Ovary Syndrome |
|  | FSH Action in Polycystic Ovary Syndrome |
|  | Glutamate Toxiicity Effects on Striatum Medium Spiny Neurons in Hantington's Disease |
|  | Bone Loss in Osteoporosis |
|  | Hyperparathyroidism, Secondary Effect |
|  | ADK Expression Downregulation after Acute Seizures |
|  | Apoptosis and Immediate Early Gene Activation in Epileptiform Disorders |
|  | Vascular Endothelial Cell Vasodilation in Brain |
|  | Nitroxyl and CGRP in Migraine Pain Activation |
|  | Dopamine Action in CSD Progression |
|  | Dopamine Synthesis and Release in Migraine |
|  | Dopamine Mediated Glutamate Release and Glutamate Uptake Circle |
|  | Vascular Smooth Muscle Cell Vasodilation in Brain |
|  | eNOS (NOS3) Activation via Prostaglandin E2 and Histamine |
|  | NO Effect on Vasodilation and Vasoconstriction |
|  | GABRB3 Mutation and Adenosine Deaminase Polymorphism |
|  | Sleep Disturbance (Hypothesis) |
|  | Prostaglandin D2 Synthase Downregulation |
|  | Local Estrogen Production in Endometriosis |
|  | LH and FSH Signaling Impairment in Primary Ovarian Insufficiency |
|  | MIF Role in Cholesteatoma |
|  | Cholesteatoma |
|  | HPV E5 Regulates EDNRA and EGFR Signaling Pathway in Keratinocytes |
|  | Netrin-1 Signaling in Age-Related Macular Degeneration |
|  | MYOC Associated Glaucoma |
|  | Noradrenalin Signaling in Glaucoma |
|  | Serotonin Signaling in the Ciliary Body |
|  | Cochlear Hair Cell Synapse Proteins Mutations (Age-Related/Congenital) |
|  | Polycystins Mutation Effects in Cystic Kidney Disease |
|  | Penile Detumescense Augmentation Mechanisms |
|  | Penile Erection Impairment |
|  | Cryptorchidism Molecular Mechanism (Hypothesis) |
|  | Leydig Cells Dysfunction in Testicular Male Infertility |
|  | Sperm Motility Impairement in Testicular Male Infertility |
|  | Sertoli Cells Dysfunction Causes Male Infertility |
|  | Ions Reabsorption Dysregulation in Nephrolithiasis |
|  | Ca2+ Reabsorption Dysregulation in Urolithiasis |
|  | Noradrenaline/Gs Expression Targets |
|  | Dopamine/Gs Expression Targets |
|  | Serotonin/Gs Expression Targets |
|  | Epinephrine/Gs Expression Targets |
|  | Acetylcholine Expression Targets |
|  | Aryl Hydrocarbon Receptor Genomic and non-Genomic Signaling |
|  | GLP1R -> ARRB1 Signaling |
|  | AGTR1 -> ARRB1/ARRB2 Signaling |
|  | GPCRs Desentization |
|  | Hedgehog Family -> ARRB1/ARRB2 Canonical Signaling |
|  | CXCL6 Expression Targets |
|  | CXCL1 Expression Targets |
|  | CXCL2 Expression Targets |
|  | CXCL3 Expression Targets |
|  | CXCL5 Expression Targets |
|  | CHRNA7 -> CREB Signaling |
|  | Complement Component GPCRs Signaling |
|  | Valdecoxib Induced Ischemic Disease |
|  | Doxorubicin Induced Cardiotoxicity |
|  | GCG Expression Targets |
|  | ADCYAP1 Expression Targets |
|  | FSHR Expression Targets |
|  | Glutamate/Gq Expression Targets |
|  | VIP Expression Targets |
|  | HIF1A in Vasculogenic Mimicry of Cancer |
|  | Myeloid Derived Suppressor Cells in Cancer Immune Escape |
|  | Adenosine/cAMP Promote Immunosuppression by Treg Cells |
|  | HCAR1/HCAR2 -> MAPK Signaling |
|  | Thromboxane A2 Expression Targets |
|  | PAF/Gq -> AP-1/ATF1/CREB/ERK/SRF Expression Targets |
|  | Dronabinol/Anandamide Expression Targets |
|  | S1P Expression Targets |
|  | PGE1 Expression Targets |
|  | MTNR1 Signaling |
|  | Purine Metabolism |
|  | OR1A1 -> GNAS Signaling |
|  | AVP/Gs -> CREB/ELK/SRF/AP-1/EGR Expression Targets |
|  | AGT -> TP53 Expression Targets |
|  | EDN1 Expression Targets |
|  | AGT -> CREB Expression Targets |
|  | SST Expression Targets |
|  | NPY Expression Targets |
|  | AGT -> ELK/SRF Expression Targets |
|  | TAC1 Expression Targets |
|  | EDN3 Expression Targets |
|  | GUCYC2 Signaling |
|  | Lysophosphatidic Acid/LPARs Signaling |
|  | Ras-GRF Regulation Signaling |
|  | AMPK Signaling |
|  | Estrogens/ESR1 non-Genomic Signaling |
|  | ERK/MAPK Canonical Signaling |
|  | Hedgehog Family Signaling |
|  | IL1B -> PGE2 Expression Targets |
|  | ROS and RNS in Vasoconstriction and Vasodilation Regulation |
|  | Model of PPARG signaling in psoriasis |
|  | Model of PPARG related pathways in psoriasis (short version) |
|  | 3\_Increased proliferation of sebocytes in acne vulgaris |
|  | 2\_Hyperseborrhea in acne vulgaris |
|  | 1\_Osteoclast-mediated bone resorption in postmenopausal women |
|  | 1\_Formation of cysts in polycystic kidney disease |
|  | 1\_Local estrogen production stimulates ectopic endometrium proliferation |
|  | 2\_Impaired steroidogenesis in PCOS |
|  | 2\_MYOC-associated glaucoma |
|  | 2\_Dysfunctions of cochlear hair cell synapse proteins in hearing loss |
|  | 1\_Endothelial cells stimulate vasoconstriction and thrombosis in pulmonary hypertension |
|  | 3\_Cardiomyocyte dysfunction unrelated to cell junctions in ARVD |
|  | 4\_Endothelial cells repress vasoconstriction in hypertension |
|  | 1\_Rise in aldosterone synthesis in adrenal zona glomerulosa cells |
|  | 2\_The intense proliferation and contractile activity of smooth muscle cells in pulmonary hypertension |
|  | 3\_1\_Mucus accumulation in asthma |
|  | 4\_2\_Airway smooth muscle cells proliferation and airway tissue remodeling |
|  | 4\_Mucin hyperproduction in goblet and mucous cells in COPD |
|  | 1\_Intestine epithelial cell dysfunction in ulcerative colitis |
|  | 3\_1\_Beta-cell proliferation and compensation: proliferation |
|  | 1\_2\_Insulin synthesis and secretion: insulin secretion |
|  | 1\_1\_Insulin synthesis and secretion: insulin processing and storage |
|  | 3\_2\_Beta-cell proliferation and compensation: neogenesis/dedifferentiation |
|  | 1\_CFTR expression and degradation in epithelial cells in cystic fibrosis (class I-II mutations) |
|  | 3\_Pain perception in diabetic neuropathy |
|  | 1\_2\_Glutamate overload and NO-mediated vasodilation |
|  | 1\_Decreased secretion of thyroid hormones |
|  | Model of PPARG signaling in psoriais (tested) |

---

|  |  |
| --- | --- |
| MedScan ID | 1216634 |

---

|  |  |
| --- | --- |
| Alias | adenosine 3,5 phosphate |
|  | adenosine-cyclic-phosphate |
|  | Adenosine 3',5'-cyclophosphorate |
|  | adenosine 3,5 monophosphate |
|  | cyclic adenosine mono-phosphate |
|  | Adenosine cyclic 3',5'-phosphate |
|  | adenosine 3',5' phosphate disodium |
|  | 24493-93-4 |
|  | cAMP |
|  | 3',5'-AMP |
|  | 3',5'-AMP sodium salt |
|  | Adenosine cyclic monophosphate |
|  | cyclic A M P |
|  | cyclic 3',5' adenosine monophosphate |
|  | 60-92-4 |
|  | 3',5' adenosine monophosphate |
|  | Acrasin |
|  | Adenosine, cyclic 3',5'-(hydrogen phosphate) |
|  | cyclic adenosyl monophosphate |
|  | Adenosine cyclic 3',5'-monophosphate |
|  | cyclic 3',5'-Adenylic acid |
|  | Cyclic 3',5'-adenylate |
|  | Adenosine 3',5'-cyclophosphate |
|  | cardimone |
|  | BRN 0052645 |
|  | Cyclic 3',5'-(hydrogen phosphate)adenosine monosodium salt |
|  | NSC 94017 |
|  | Adenosine 3,5'-cyclic monophosphoric acid |
|  | (S)-1-C-(7-Amino-1H-pyrazolo(4,3-d)pyrimidine-3-yl)-1,4-anhydro-D-ribitol cyclic 3',5'-(hydrogen phosphate) |
|  | 3',5'-cyclic AMP |
|  | adenosine, cyclic 3',5'-(hydrogenphosphate) |
|  | Adenosine 3',5'-monophosphate |
|  | cyclic adenylate |
|  | cyclic AMP sodium |
|  | cyclic-3',5'-(hydrogen phosphate)adenosine |
|  | adenosine-3',5'-monophosphorate |
|  | cyclic 3',5' adenosine phosphate |
|  | cyclic 3',5'-AMP |
|  | cyclic Adenosine 3',5'-phosphate |
|  | EINECS 200-492-9 |
|  | CCRIS 4291 |
|  | cyclic adenosine monophosphate |
|  | 11002-78-1 |
|  | 6-(6-Amino-9H-purine-9-yl)tetrahydro-4H-furo[3,2-d][1,3,2]dioxaphosphinine-2,7-diol 2-oxide |
|  | Adenosine 3,5'-cyclic monophosphorate |
|  | 37839-81-9 |
|  | Adenosine-3',5'-cyclic phosphate |
|  | 3',5'-cyclic adenosine monophosphate |
|  | 85187-54-8 |
|  | adenosine 3',5' monophosphate monohydrate |
|  | cyclic adenosine 3,5 phosphate |
|  | adenosine-3',5'-monophosphoric acid |
|  | cyclic adenylic acid |
|  | cyclic AMP |
|  | formycin cyclic 3',5'-monophosphate |
|  | Adenosine 3',5'-cyclophosphoric acid |
|  | Adenosine 3',5'-cyclic monophosphate |
|  | cyclic AMP disodium |
|  | adenosine 3,5 cyclic monophosphate |
|  | cyclic 3,5 adenosine monophosphate |
|  | cyclic Adenosine 3',5'-monophosphate |
|  | adenyl cyclate |
|  | Cyclic 3',5'-AMP sodium salt |
|  | mono phosaden |
|  | adenosine-cyclic-phosphoric-acid |
|  | adenosine 3',5' phosphoric acid |
|  | Adenosine 3',5'-phosphate monohydrate |
|  | adenosine 3,5 cyclic phosphate |
|  | cyclic 3,5-AMP |
|  | 54532-48-8 |
|  | cyclic adenosine phosphate |
|  | 3,5 adenosine monophosphate |
|  | adenosine 3,5 cyclic monophosphoric acid |
|  | adenosine 3,5 phosphoric acid |
|  | c-AMP |
|  | cyclic adenosine 3,5 monophosphate |
|  | Adenosine 3',5'-phosphate |
|  | 6-(6-Amino-9H-purin-9-yl)tetrahydro-4H-furo[3,2-d][1,3,2]dioxaphosphinine-2,7-diol 2-oxide |
|  | cyclic 3,5 amp |
|  | adenosine 3', 5'-phosphate |

---

|  |  |
| --- | --- |
| CAS ID | 60-92-4 |
|  | 11002-78-1 |
|  | 54532-48-8 |
|  | 37839-81-9 |
|  | 85187-54-8 |

---

|  |  |
| --- | --- |
| Reaxys ID | 10105600 |
|  | 1041600 |
|  | 1229040 |
|  | 1229041 |
|  | 1229042 |
|  | 1229043 |
|  | 1229044 |
|  | 18639706 |
|  | 3631062 |
|  | 4650157 |
|  | 4650158 |
|  | 5215289 |
|  | 52645 |
|  | 9220381 |
|  | 9361226 |
|  | 965119 |

---

|  |  |
| --- | --- |
| ChEBI ID | 17489 |

---

|  |  |
| --- | --- |
| HMDB ID | HMDB00058 |

---

|  |  |
| --- | --- |
| KEGG ID | C00575 |

---

|  |  |
| --- | --- |
| InChIKey | IVOMOUWHDPKRLL-KQYNXXCUSA-N |
|  | BXJBFCKTIWRKMQ-MCDZGGTQSA-M |
|  | IFHPFJUCYLXWOT-KSYZLYKTSA-N |
|  | IVOMOUWHDPKRLL-UHFFFAOYSA-N |

---

|  |  |
| --- | --- |
| Molecular Formula | C10H12N5O6P |
|  | C10H11N5NaO6P |

---

|  |  |
| --- | --- |
| PubChem SID | 134970712 |
|  | 135228313 |
|  | 135258346 |
|  | 148964 |
|  | 789944 |
|  | 3778229 |
|  | 3882616 |

---

|  |  |
| --- | --- |
| PubChem CID | 6076 |
|  | 118704577 |
|  | 23669773 |
|  | 274 |

---

|  |  |
| --- | --- |
| XLogP-AA | -2.6 |
|  | -2.9 |

---

|  |  |
| --- | --- |
| IUPAC Name | (4aR,6R,7R,7aS)-6-adenin-9-yl-2-hydroxy-2-keto-4a,6,7,7a-tetrahydro-4H-furo[3,2-d][1,3,2]dioxaphosphorin-7-ol |
|  | sodium;(4aR,6R,7R,7aS)-6-adenin-9-yl-2-keto-2-oxido-4a,6,7,7a-tetrahydro-4H-furo[3,2-d][1,3,2]dioxaphosphorin-7-ol |
|  | (4aR,6S,7S,7aS)-6-(7-amino-2H-pyrazolo[4,3-d]pyrimidin-3-yl)-2-hydroxy-2-keto-4a,6,7,7a-tetrahydro-4H-furo[3,2-d][1,3,2]dioxaphosphorin-7-ol |
|  | 6-adenin-9-yl-2-hydroxy-2-keto-4a,6,7,7a-tetrahydro-4H-furo[3,2-d][1,3,2]dioxaphosphorin-7-ol |
|  | sodium (4aR,6R,7R,7aS)-6-adenin-9-yl-2-keto-2-oxido-4a,6,7,7a-tetrahydro-4H-furo[3,2-d][1,3,2]dioxaphosphorin-7-ol |

---

|  |  |
| --- | --- |
| Rotatable Bond Count | 1 |

---
